# Effects of the Interactive Web-Based Video “Mon Coeur, Mon BASIC” on Drug Adherence of Patients With Myocardial Infarction: Randomized Controlled Trial

**DOI:** 10.2196/21938

**Published:** 2021-08-30

**Authors:** Christel Bruggmann, Julien Adjedj, Sylvain Sardy, Olivier Muller, Pierre Voirol, Farshid Sadeghipour

**Affiliations:** 1 Department of Pharmacy University Hospital of Lausanne University of Lausanne Lausanne Switzerland; 2 Institute of Pharmaceutical Sciences of Western Switzerland University of Geneva Geneva Switzerland; 3 Department of Pharmacy University Hospital of Geneva Geneva Switzerland; 4 Department of Cardiology University Hospital of Lausanne University of Lausanne Lausanne Switzerland; 5 Section of Mathematics University of Geneva Geneva Switzerland

**Keywords:** acute coronary syndrome, eHealth, drug adherence, mHealth, mobile phone

## Abstract

**Background:**

Secondary prevention strategies after acute coronary syndrome (ACS) presentation with the use of drug combinations are essential to reduce the recurrence of cardiovascular events. However, lack of drug adherence is known to be common in this population and to be related to treatment failure. To improve drug adherence, we developed the “Mon Coeur, Mon BASIC” video. This online video has been specifically designed to inform patients about their disease and their current medications. Interactivity has been used to increase patient attention, and the video can also be viewed on smartphones and tablets.

**Objective:**

The objective of this study was to assess the long-term impact of an informative web-based video on drug adherence in patients admitted for an ACS.

**Methods:**

This randomized study was conducted with consecutive patients admitted to University Hospital of Lausanne for ACS. We randomized patients to an intervention group, which had access to the web-based video and a short interview with the pharmacist, and a control group receiving usual care. The primary outcome was the difference in drug adherence, assessed with the Adherence to Refills and Medication Scale (ARMS; 9 multiple-choice questions, scores ranging from 12 for perfect adherence to 48 for lack of adherence), between groups at 1, 3, and 6 months. We assessed the difference in ARMS score between both groups with the Wilcoxon rank sum test. Secondary outcomes were differences in knowledge, readmissions, and emergency room visits between groups and patients’ satisfaction with the video.

**Results:**

Sixty patients were included at baseline. The median age of the participants was 59 years (IQR 49-69), and 85% (51/60) were male. At 1 month, 51 patients participated in the follow-up, 50 patients participated at 3 months, and 47 patients participated at 6 months. The mean ARMS scores at 1 and 6 months did not differ between the intervention and control groups (13.24 vs 13.15, 13.52 vs 13.68, respectively). At 3 months, this score was significantly lower in the intervention group than in the control group (12.54 vs 13.75; *P*=.03). We observed significant increases in knowledge from baseline to 1 and 3 months, but not to 6 months, in the intervention group. Readmissions and emergency room visits have been very rare, and the proportion was not different among groups. Patients in the intervention group were highly satisfied with the video.

**Conclusions:**

Despite a lower sample size than we expected to reach, we observed that the “Mon Coeur, Mon BASIC” web-based interactive video improved patients’ knowledge and seemed to have an impact on drug adherence. These results are encouraging, and the video will be offered to all patients admitted to our hospital with ACS.

**Trial Registration:**

ClinicalTrials.gov NCT03949608; https://clinicaltrials.gov/ct2/show/NCT03949608

## Introduction

Cardiovascular disease is a major cause of morbi-mortality in Europe, with a substantial contribution from acute coronary syndrome (ACS) [1]. Secondary prevention strategies, such as risk factor control through lifestyle modifications and the use of medication combinations, have greatly reduced the recurrence of ACS [2]. European guidelines have been developed to enhance evidence-based medicine prescriptions for patients with myocardial infarction (MI) [3,4], and we previously showed that physicians at our hospital in Switzerland predominantly issued prescriptions of this type [5]. However, poor patient self-adherence to cardiac medications has been documented worldwide [6-8] and has been associated with increased morbi-mortality [9-11]. The discontinuation of antiplatelet drugs has been related to fatal consequences such as stent thrombosis, particularly soon after ACS onset [12].

Poor drug adherence is related to many factors associated with health care systems (eg, cost, access to care), socioeconomics (ie, poverty), therapy (ie, treatment complexity, cost), and patients (ie, health literacy, willingness to change, knowledge, education) [13]. In Switzerland, the health care system offers high-quality care to all residents, and less social inequality exists than in other countries; we thus believe that patient adherence in this country is more likely related to treatment and patient factors. The provision of sufficient and effective information to patients with chronic diseases has been shown to increase patient satisfaction [14], reduce psychological distress [15], enhance patients’ perceived control [16], and improve patient adherence to medication prescriptions [13]. Thus, the offering of such information and knowledge to all patients with ACS is very important.

Various interventions have been shown to promote drug adherence in the context of cardiovascular health. These strategies range from the simple, such as the distribution of written material about medications [17] and the regular mailing of informational letters [18,19], to multifaceted interventions (eg, medication reconciliation, therapeutic education) involving clinical pharmacists [20,21] and nurses specialized in therapeutic education [22].

The length of hospital stays after MI has shortened in recent years, which has reduced the number of opportunities to inform patients about their disease during hospitalization. Cardio-rehabilitation (CR) centers are meant to fill this gap, but not all patients participate in CR programs. In addition, depending on the type of program chosen (ie, stationary or ambulatory), the objective may be oriented more toward cardiovascular reeducation than to patient education. Moreover, very few sessions are devoted to educating patients about their drug treatments.

Different kinds of mobile health (mHealth) technologies have been developed to support cardiovascular health, like eHealth diaries [23], apps supporting cardiac rehabilitation [24,25], and complete e-learning platforms [26]. However, none of these technologies were available in French, and only a few studies have evaluated the impact of mHealth technologies on therapeutic adherence [27,28]. As we were convinced that mHealth technologies have an impact on drug adherence, we wanted to test it with our study. Therefore, we developed a new approach using a video tool for the provision of information and patient education. The tool we developed is interactive, web-based, smartphone- and tablet-compatible, and it is designed to be offered to patients with ACS during their hospital stays. We tested whether this tool has impacts on drug adherence and patient knowledge after ACS. Our study is therefore expected to fill some important gaps in the current literature.

## Methods

### Study Design and Population

The Secondary Prevention of ACS With Beta-Blockers, Antiaggregants, Statins, Angiotensin-Converting Enzyme Inhibitors, and Risk Factor Control (BASIC) study was a single-center randomized trial. The objective of the study was to assess if a new web-based and interactive video could increase long-term drug adherence, as well as the knowledge of the patients about their disease and their current medications, in patients admitted in hospital for an ACS.

The patients were screened between February 1 and September 1, 2019, on admission to the University Hospital of Lausanne. The intervention for this study was added to usual care, in which all patients with ACS watched a 20-minute video called ELIPS during their hospital stays [29]. ELIPS explains acute infarction, acute care, and the drugs prescribed. Patients also received a booklet about coronary artery disease. All patients admitted to our hospital for ACS are encouraged to participate in a CR program after discharge; the program chosen depends on each patient’s willingness and home location. Two types of programs are offered (a 3-week stationary program and an ambulatory program), and both are reimbursed by health insurance.

Participants eligible for the study were men and women older than 18 years who were diagnosed with ST-segment elevation myocardial infarction (STEMI) or non–ST-segment elevation myocardial infarction (NSTEMI) treated with percutaneous coronary intervention (PCI). Other requirements were total discernment capacity; possession of a digital tablet, smartphone, or home computer; and satisfactory French language skills. Exclusion criteria were the inability to follow the study procedure (eg, due to language problems, psychological disorders, dementia), refugee claimant status, homelessness or incarceration (because of the impossibility of contacting such individuals after discharge), and life expectancy <6 months due to another disease.

Eligible participants were randomized to the intervention and control groups using a 1:1 allocation ratio after providing oral and written informed consent. The randomization was conducted by a clinical study specialist who was not involved in the study. The randomization process was made by week and block of 4. Each week was randomized as intervention or control in order to avoid having two patients in different groups next to each other in the intermediate care unit.

### “Mon Coeur, Mon BASIC” Web-Hosted Video

**Figure 1:**
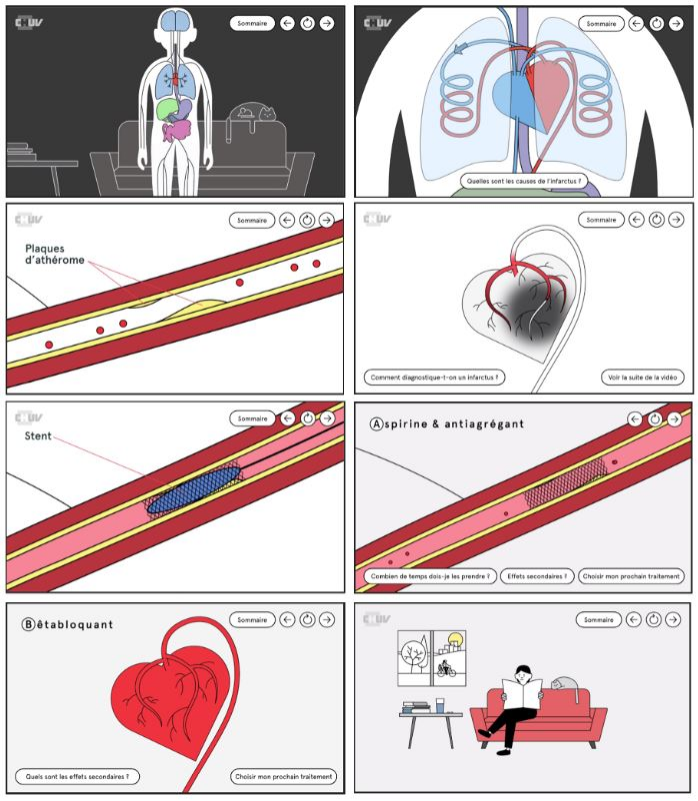
Screenshots from the “Mon Coeur, Mon BASIC” video.

“Mon Coeur, Mon BASIC” is an interactive web-hosted video that is smartphone- and tablet-compatible. It is freely available online [30]. It is a simple animated cartoon (Figure 1) with narration in French. It consists of three parts providing information about heart function and the physiopathology of ACS, acute care for ACS (coronarography and PCI), and the medications prescribed after ACS (usefulness and side effects), with a total length of about 15 minutes. The viewer can click on the video using a mouse or finger to obtain information about a particular point or to jump directly to another subject. The video was developed by a working group consisting of a pharmacist, a graphic designer, a specialist in patient communication, and a cardiologist. It was designed using Illustrator and InDesign (both from Adobe Inc, San José, California), and the interactivity was created using Storyline 360 (Articulate, New York). It was tested with several patients, and all bugs were eliminated before the beginning of the study.

### Intervention and Control Condition

The pharmacist participating in the study met all patients in the intervention group during their hospital stays and gave each of them a medication card with his or her current prescriptions (Multimedia Appendix 1). The medication card contained all the medications currently prescribed to the patient, classified by type of medication according to the acronym BASIC for Beta-blocker, Antiaggregant, Statin, angiotensin-converting enzyme Inhibitor, and Control of risk factors (ie, smoke cessation, limitation of fatty meals, etc), and was the size of a credit card. She then connected to the e-learning website on the patient’s tablet or smartphone and created a shortcut. Tablets from the institution were lent to patients with no tablet, smartphone, or computer. The pharmacist asked the patients to watch the video at their convenience. After the patients had watched the video, the pharmacist visited to answer questions and distribute study questionnaires (described below). The postvideo visit was short, and the aim of the visit was to check if the patients had watched the video and to answer patients’ questions if they had any. The duration of this visit was around 10 minutes per patient.

The control group received usual care. In usual care, the patient has no meeting with the pharmacist at all, and the information about medications and the disease are provided by the nurses and the physician in charge of the patient during hospitalization. All the patients (in control and intervention group) also watched a short film developed in the Geneva University Hospitals [29]. For the purpose of the study, the pharmacist only met patients randomized to the control group to give them the study questionnaires.

### Primary Outcome

The primary outcome was the difference in medication adherence between groups at 1, 3, and 6 months after ACS for the assessment of postdischarge treatment initiation, treatment implementation in daily life, and long-term treatment persistence, respectively. Adherence was assessed using the Adherence to Refills and Medication Scale (ARMS; Multimedia Appendix 2). This self-report questionnaire was chosen due to its strong internal consistency (Cronbach α=.814), good correlation with other subjective and objective measures, and validation for use with patients with coronary heart disease [32]. It consists of 12 items assessing adherence to taking medication (n=8) and refilling prescriptions (n=4). The questionnaire is made up of affirmations that can be answered with “none of the time,” “some of the time,” “most of the time,” or “all of the time,” varying from 1 to 4 points, respectively. Final scores range from 12 (most adherence) to 48 (least adherence) and can be treated as continuous measures or dichotomized as 12 and >12. We translated the validated English version of the ARMS into French according to guidelines developed for health care research [33].

### Secondary Outcomes

The first secondary outcome was the difference in ACS-related knowledge (basics of heart function, ACS pathophysiology, and usefulness of prescribed medications) between groups, assessed using a questionnaire developed specifically for this study (Multimedia Appendix 3). The questionnaire has 9 items, and scores range from 0 to 9 (most knowledge). The questionnaire was given to the intervention group as a pretest (before the video viewing) and posttest (after viewing), to the control group 1 day after study inclusion, and to all participants at 1, 3, and 6 months. We also assessed whether the groups differed in a composite measure of cardiovascular mortality, first occurrence of reinfarction, recurrence of ACS, cardiovascular death, and readmission and emergency room visitation over the 6-month study period. Finally, we assessed patients’ satisfaction with the video using a questionnaire of 11 multiple-choice questions.

### Data Collection

Most baseline data (eg, demographic characteristics, laboratory values, and vital parameters on day 2 after ACS; ACS type and therapeutic strategy; cardiovascular risk factors; drug prescribed at discharge) were collected from computerized patient records generated during hospitalization. Other data were collected through patient interviews; they included patients’ email addresses, educational levels (graduation of primary, secondary, or tertiary school), employment statuses (full time, part time, retired, or unemployed), spoken French levels (native, near native, highly proficient, very good working knowledge, or basic communication skills), general practitioners’ names, types of device used at home (smartphone, tablet, or computer), information and communications technology (ICT) use levels (low: short message service or telephone only, medium: also maps and basic online research, or high: many applications in daily life), and health literacy scores, assessed with the validated French translation of the Functional, Communicative and Critical Health Literacy (FCCHL) tool [34]. FCCHL scores range from 14 (least literacy) to 70 (most literacy). All data were collected and managed using REDCap (Research Electronic Data Capture) tools hosted in the University Hospital of Lausanne [35,36]. REDCap is a secure, web-based software platform designed to support data capture for research studies.

An email with a link to the study questionnaires (hosted by REDCap) was sent to the participants at 1, 3, and 6 months after study inclusion. For participants who did not provide any email address, the questionnaires were mailed with postage-paid return envelopes.

### Statistical Analysis

An intention-to-treat analysis including all study participants was performed. The characteristics of patients randomized to the intervention and control groups were expressed as medians with 25th and 75th percentiles for continuous variables, and as numbers and percentages for categorical variables. They were compared using the Pearson chi-square test for categorical variables and the *t* test for continuous variables.

The ARMS scores at 1, 3, and 6 months were compared between groups using the Wilcoxon rank sum test. This test was also used to compare pretest and posttest knowledge scores and those at 1, 3, and 6 months between groups. The Wilcoxon rank sum test for paired data was used to assess differences in knowledge within groups between study timepoints. Descriptive statistics (numbers and percentages) were calculated for the composite outcome, satisfaction scores, and video reuse. The level of significance for all analyses was set at two-sided α<.05. All analyses were performed using Stata software (version 14; StataCorp, College Station, Texas).

We performed a power analysis to estimate the sample size required to detect a significant difference in medication adherence between the intervention and control groups. We based the calculation on the mean ARMS score of 16.32 (SD 4.06) for chronically ill patients [32]. We determined that a sample of 128 patients (64 per group) was needed to detect a difference of 0.5 SD (ie, 2.03) between the intervention and control groups at a two-sided 5% significance level with a power of 0.8 and an allocation ratio of 1:1. Assuming 10% loss to follow-up, the target sample size was 142 patients. Based on our hospital’s annual admission rate of 500 patients with NSTEMI and 300 patients with STEMI, we initially believed that study enrollment would be completed within 20 weeks, but because many patients were discharged before we could conduct baseline assessment, and an unexpectedly large number of patients refused to participate, we extended the enrollment period to 37 weeks. However, because the investigator was not available for a longer period of time than planned, we had to terminate the study before the target sample size had been reached. The results presented in this article must therefore be interpreted with caution because the study was underpowered.

### Ethical Considerations

This study was approved by the local ethics committee (*Commission cantonale d’éthique de la Recherche sur l’être humain du Canton de Vaud*; number 2018-02223) and registered at ClinicalTrials.gov (number NCT03949608). It complied with the principles of good clinical practice and the Declaration of Helsinki.

## Results

### Study Population and Baseline Characteristics

**Figure 2:**
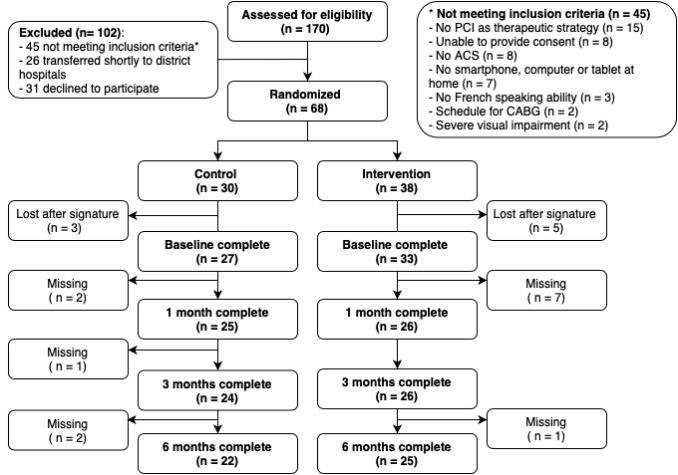
Patient flowchart within the study period. ACS: acute coronary syndrome; CABG: coronary artery bypass grafting; PCI: percutaneous coronary intervention.

In total, 170 patients were screened during the study period, and 125 patients were asked to participate (Figure 2). Of these, 31 (24.8%) patients declined participation, and 26 (20.8%) were transferred to district hospitals shortly thereafter and were not enrolled. We randomized 68 patients to the control (n=30) and intervention (n=38) groups. Of these, 8 patients were transferred unexpectedly to district hospitals, did not watch the video, or did not complete the baseline questionnaires; thus, the final baseline sample consisted of 60 patients (33 in the intervention group and 27 in the control group) with complete baseline data. An additional 21 of the 68 randomized patients (31%) did not complete the follow-up questionnaires.

The median age of the 60 patients included was 59 years; 85% (51/60) of the patients were men, 19% (11/59) had diabetes, and about 45% (25/56) were active smokers (Table 1). Patients in the control group were slightly older and more often retired than patients in the intervention group were. They also had more comorbidities and more often had NSTEMI than did patients in the intervention group. However, the groups were well matched, with no significant difference in baseline characteristics.

**Table 1:** Baseline patient characteristics (N=60). Categorical data are presented as n (%) and continuous data are presented as median (interquartile range). *P* values were estimated with the chi-square test for categorical variables and *t* test for continuous variables.

Characteristic				Overall (N=60)		Intervention (n=33)		Control (n=27)		*P* value		
**Demographic characteristics**												
	Sex (male), n (%)			51 (85)		27 (82)		24 (89)		.45		
	Age (years), median (IQR)			59 (49-69)		56 (46-68)		62 (56-71)		.10		
	**Civil status, n (%)**										.70	
		Single	10 (17)		4 (12)		6 (22)					
		Married	36 (60)		20 (61)		16 (59)					
		Divorced	11 (18)		7 (21)		4 (15)					
		Widowed	3 (5)		2 (6)		1 (4)					
	**Education level, n (%)**									.54		
		Compulsory school	8 (13)		6 (18)		2 (7)					
		High school, internship	28 (47)		15 (45)		13 (48)					
		University of applied sciences	8 (13)		3 (9)		5 (18.5)					
		Bachelor’s degree	5 (8)		2 (6)		3 (11.1)					
		Master’s degree or more	11 (18)		7 (21)		4 (14.8)					
	**Employment, n (%)**									.17		
		Full time	29 (48)		20 (61)		9 (33.3)					
		Part time	7 (12)		2 (6)		5 (19)					
		Retired	20 (33)		9 (27)		11 (41)					
		Unemployed	4 (7)		2 (6)		2 (7)					
	**French speaking level, n (%)**									.66		
		Native	48 (80)		26 (79)		22 (81)					
		Near native	12 (20)		7 (21)		5 (19)					
	**Device type, n (%)**											
		Computer	55 (92)		30 (91)		25 (93)		.81			
		Smartphone	51 (85)		28 (85)		23 (85)					
		Tablet	38 (63)		24 (73)		14 (52)					
	**ICT^a^ use level, n (%)**									.37		
		Low	12 (20)		7 (21)		5 (19)					
		Medium	9 (15)		3 (9)		6 (22)					
		High	39 (65)		23 (70)		16 (59)					
	Health literacy (FCCHL^b^) score, median (IQR)			49 (44.5-54)		50 (44-54)		48 (45-54)		.95		
**Cardiovascular risk factors**												
	**Smoking status^c^, n (%)**									.13		
		Active	25 (45)		14 (47)		11 (42)					
		Former	17 (30)		9 (30)		8 (31)					
	Diabetes^d^, n (%)			11 (19)		5 (16)		6 (22)		.52		
	Hypertension, n (%)			32 (53)		17 (52)		15 (56)		.76		
	Dyslipidemia^e^, n (%)			34 (59)		23 (72)		11 (42)		.02		
	Alcohol consumption^f^, n (%)			5 (10)		3 (9)		2 (11)		.89		
	Family history of ACS^g,h^, n (%)			24 (44)		15 (47)		9 (41)		.67		
	Overweight, n (%)			37 (62)		21 (64)		16 (59)		.73		
	Drugs used chronically before ACS, median (IQR)			1 (0-3)		0 (0-2)		2 (1-3)		.12		
**ACS type, n (%)**												
	STEMI^i^			52 (87)		31 (94)		21 (78)		.07		
	NSTEMI^j^			8 (13)		2 (6)		6 (22)				
**LVEF^k^ evaluation during hospitalization, n (%)**												.23
	<40%			6 (10)		2 (6)		4 (15)				
	≥40%			54 (90)		31 (94)		23 (85)				
**Discharge**												
	Total length of stay (days), median (IQR)			3 (2-4)		3 (2-4)		3 (2-4)		.70		
	**Destination after university hospital discharge, n (%)**										.71	
		Home	40 (67)		23 (70)		17 (63)					
		Cardio-rehabilitation center	2 (3)		1 (3)		1 (4)					
		District hospital	17 (28)		8 (24)		9 (33)					
		Another in-hospital department	1 (2)		1 (3)		0 (0)					
	Drugs prescribed at discharge, median (IQR)			6 (6-7.5)		6 (6-7)		7 (5-9)		.53		
	**Prescriptions at discharge, n (%)**											
		Beta-blocker	53 (88)		27 (82)		26 (96)		.08			
		ACEI^l^	55 (92)		30 (91)		25 (93)		.81			
		Statin	59 (98)		33 (100)		26 (96)		.27			
		Aspirin	60 (100)		33 (100)		27 (100)		N/A^m^			
		P2Y_12_ inhibitor	60 (100)		33 (100)		27 (100)		N/A			

^a^ICT: information and communications technology.

^b^FCCHL: Functional, Communicative and Critical Health Literacy tool (French translation).

^c^Four missing values (3 in the intervention group, 1 in the control group).

^d^One missing value (intervention group).

^e^Two missing values (1 in each group).

^f^Nine missing values (1 in the intervention group, 8 in the control group).

^g^Six missing values (1 in the intervention group, 5 in the control group).

^h^ACS: acute coronary syndrome.

^i^STEMI: ST-segment elevation myocardial infarction.

^j^NSTEMI: non–ST-segment elevation myocardial infarction.

^k^LVEF: left ventricular ejection fraction.

^l^ACEI: angiotensin-converting enzyme inhibitor.

^m^N/A: not applicable.

### Primary Outcome: ARMS Score

**Figure 3:**
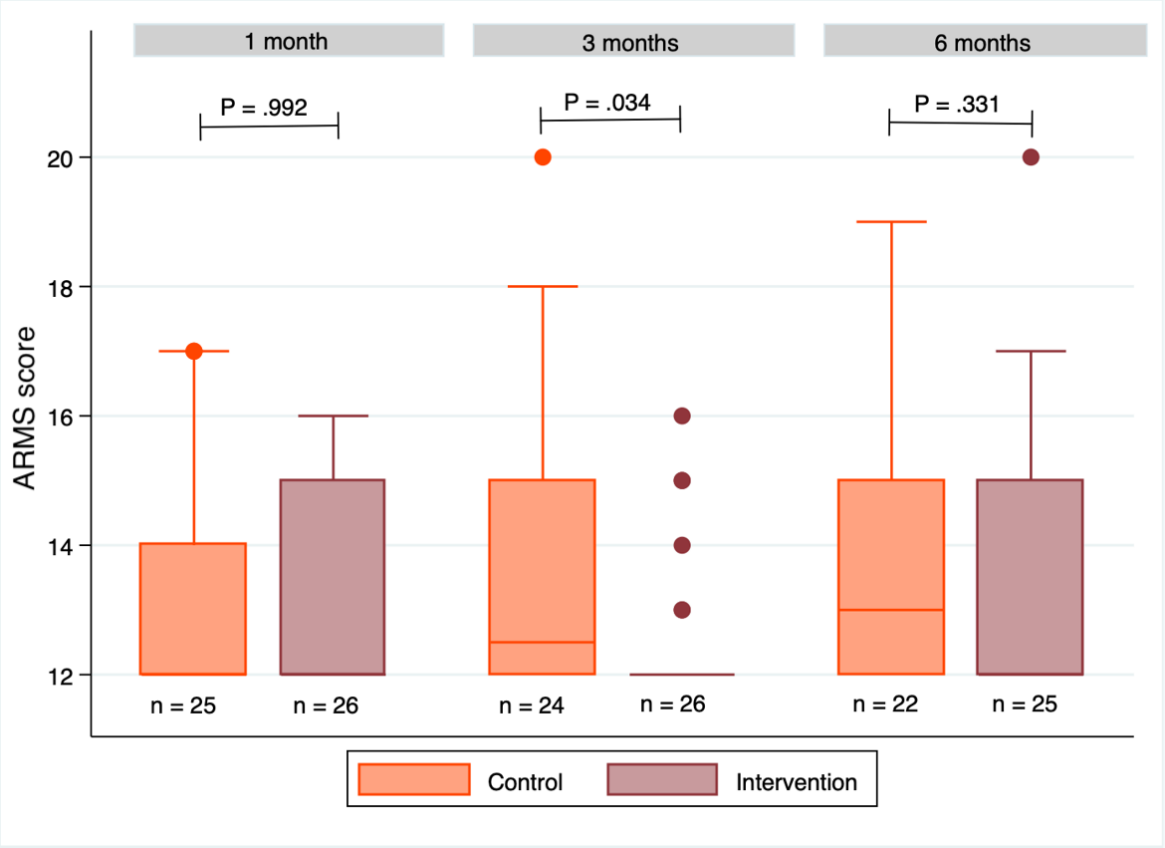
ARMS scores at 1, 3, and 6 months. In the box plots, the boundary of the box closest to zero indicates the 25th percentile, the line within the box represents the median, and the boundary of the box farthest from zero indicates the 75th percentile. Whiskers above the box indicate the 90th percentile. Points above the whiskers represent the outliers outside the 90th percentile. The *P* values represent statistics made with the nonparametric Wilcoxon test. n represents the number of participants per group. ARMS: Adherence to Refills and Medication Scale.

At 1 month, the mean ARMS score did not differ significantly between the intervention and control groups (13.15, 95% CI 12.56-13.74 and 13.24, 95% CI 12.52-13.96, respectively; *P*=.99). At 3 months, the ARMS score was significantly lower in the intervention group than in the control group (12.54, 95% CI 12.08-13.00 vs 13.75, 95% CI 12.74-14.76; *P*=.03). At 6 months, this score did not differ between the intervention and control groups (13.52, 95% CI 12.63-14.41 and 13.68, 95% CI 12.96-14.76, respectively; *P*=.33; Figure 3). The median ARMS score increased from 1 to 6 months in the control group but remained more stable (at ~12) in the intervention group, despite a change in distribution.

Of 13 participants whose ARMS scores increased between 1 and 3 months, 10 were in the intervention group, and 3 were in the control group. In 7 (54%) cases, scores increased because patients provided different responses to the last item about prescription refills (Multimedia Appendix 2).

### Secondary Outcomes

#### Knowledge Score

**Figure 4:**
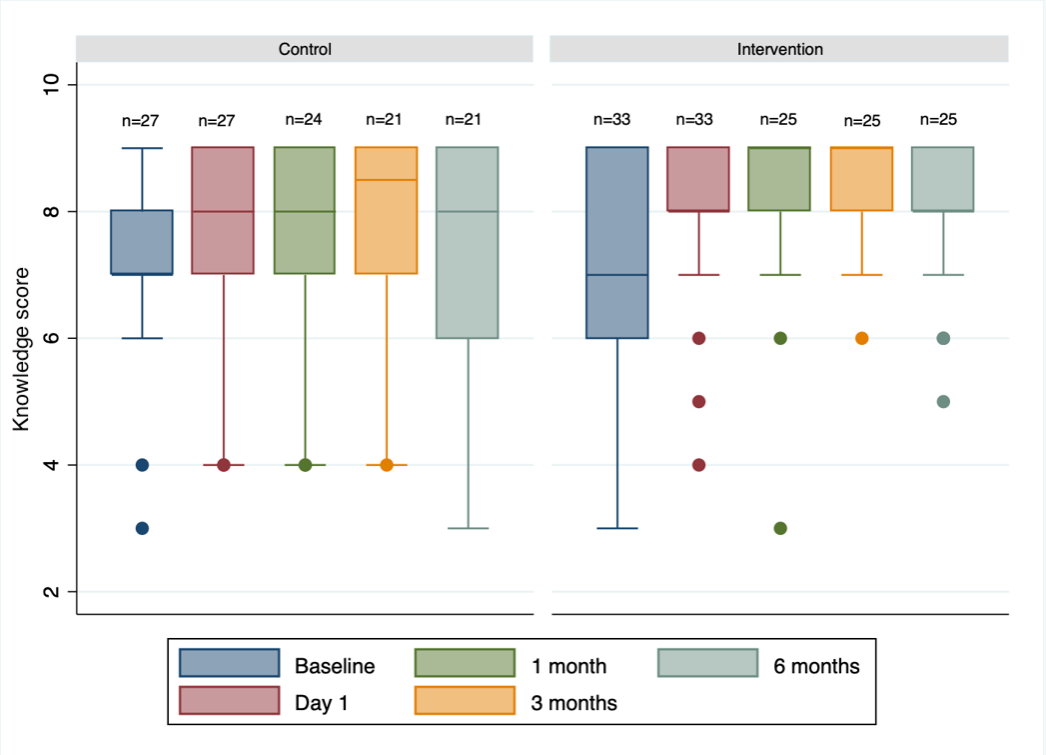
Knowledge scores (0-9) at baseline (pretest), 1 day after ACS (posttest), and 1, 3, and 6 months after ACS. In the box plots, the boundary of the box closest to zero indicates the 25th percentile, the line within the box represents the median, and the boundary of the box farthest from zero indicates the 75th percentile. Whiskers above the box indicate the 90th percentile. Points above the whiskers represent the outliers outside the 90th percentile. n represents the number of participants per group. ACS: acute coronary syndrome.

The mean knowledge score did not differ significantly between the intervention and control groups at baseline or at 1, 3, or 6 months (7.22, 95% CI 6.64-7.81 and 7.03, 95% CI 6.46-7.60; 8.19, 95% CI 7.66-8.72 and 7.72, 95% CI 7.06-8.38; 8.36, 95% CI 8.03-8.69 and 8.00, 95% CI 7.44-8.56; and 8.04, 95% CI 7.54-8.54 and 7.72, 95% CI 7.00-8.45, respectively; all *P*s>.05; Figure 4). Within each group, knowledge increased from baseline to 6 months; increases were significant in the intervention group between baseline and 3 months, but not at 6 months (Table 2).

**Table 2:** Differences in knowledge scores from baseline.

Timepoint		Control, median (95% CI)	*P* value^a^	Intervention, median (95% CI)	*P* value^a^
Baseline score		7.22 (6.64 to 7.81)	N/A^b^	7.03 (6.46 to 7.60)	N/A
**Difference from baseline**					
	Posttest (1 day)	0.33 (–0.16 to 0.82)	.14	1.00 (0.48 to 1.52)	.001
	1 month	0.48 (–1.13 to 1.19)	.17	1.04 (0.35 to 1.73)	.03
	3 months	0.63 (–0.02 to 1.27)	.06	1.40 (0.68 to 2.12)	.008
	6 months	0.32 (–0.30 to 0.94)	.27	0.88 (0.25 to 1.51)	.144

^a^Wilcoxon rank sum test for paired data.

^b^N/A: not applicable.

#### Composite Endpoint of Mortality, Reinfarction, Rehospitalization, and Emergency Room Visits

No death occurred in the cohort. Five patients visited the emergency room and were subsequently hospitalized. Overall, 7 of 46 (15%) patients (2 in the intervention group, 5 in the control group) were hospitalized for cardiovascular reasons (heart rhythm disorders: 1 tachycardia, 1 bradycardia; vagal discomfort: n=2; chest pain that was determined to be noncardiac: n=1; elective stent placement: n=1; and coronary artery bypass grafting: n=1). No patient had a new infarct during the follow-up period and no difference was observed between groups.

#### Satisfaction With and Reuse of the Video and Medication Card

**Figure 5:**
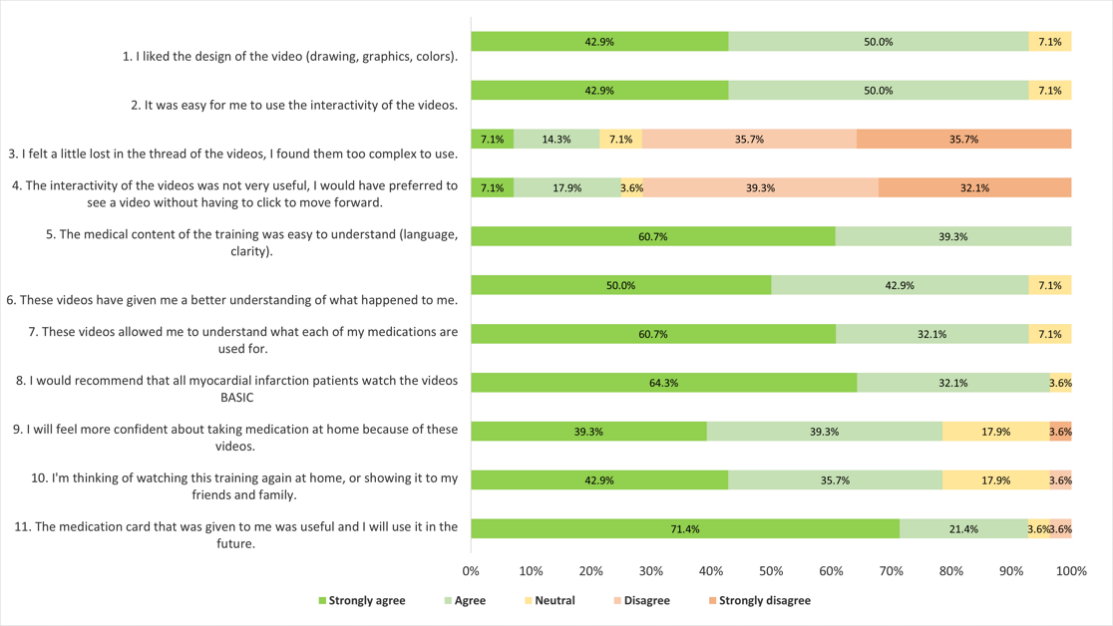
Patients’ satisfaction with the video (n=28).

Overall, the patients were highly satisfied with the design, use, and medical content of the video (Figure 5). The majority of patients felt that the video helped them to better understand their disease and medications and indicated that they would recommend it to other patients with ACS. As questions 3 and 4 were negatively worded, some patients may have answered them incorrectly. Despite this potential issue, the majority of patients felt comfortable with the interactivity of the video and did not lose the thread of the presentation. Responses regarding whether patients thought they would watch the video again at home were the most mitigated. We found that 18 of 30 (60%) patients viewed the video again at home. The majority of patients strongly appreciated the medication cards, and 22 of 30 (73%) patients reused the cards after discharge.

## Discussion

### Principal Findings

The “Mon Coeur, Mon BASIC” web-hosted video seemed to significantly improve the drug adherence of patients with ACS for a few months after treatment initiation: a significant effect was observed at 3 months, but not at 1 or 6 months. Moreover, patients from the intervention group showed significant improvement in their knowledge about ACS and their medications from baseline to 3 months; no such effect was seen in the control group. In addition, patients were generally very satisfied with the information provided in the video and with the medication cards given to them.

The rate of drug adherence was high in both groups at 1 month after ACS. As expected, the median ARMS score tended to increase over time in the control group, reflecting decreasing adherence. At 3 months, the intervention group showed much better drug adherence than did the control group. At 6 months, the median score remained lower in the intervention group, but this difference was not significant. Although the observed trends may not all be attributable to the effect of the video alone, we can state with strong confidence that patients in the intervention group were more likely to refill their prescriptions at 3 months than at 1 month.

In the intervention group, patients’ knowledge about ACS and their medications increased significantly from baseline to 1 day (after video viewing) and 3 months after ACS. Thus, the intervention appeared to effectively improve patients’ cardiovascular knowledge over the first 3 months after ACS presentation.

Overall, our results are encouraging but must be interpreted with caution. The sample size initially calculated could not be reached, and our study is therefore underpowered. However, our results are still interesting and show a great tendency for improvement of drug adherence and knowledge.

### Comparison With Previous Work

App use has been demonstrated to improve drug adherence. For example, use of the “My Interventional Drug-Eluting Stent Education” app, developed in the United States with a similar goal as the “Mon Coeur, Mon BASIC” video, increased patients’ antiplatelet drug adherence and knowledge about PCI in a pilot study [27]. Use of another patient support app increased ticagrelor adherence and tended to improve cardiovascular-related lifestyle changes [28]. mHealth technologies seem to have an impact in drug adherence and should be part of future developments in the cardiology field.

### Place of mHealth Technology for ACS Patients

Most currently available mHealth technologies were developed to support cardiovascular health; products include eHealth diaries [23], apps supporting cardiac rehabilitation [24,25], and complete e-learning platforms [26]. We are convinced that these strategies will make up a large part of future patient care, but their use will likely remain limited for a large proportion of current cardiac patients. The median age of our patients at admission was 59 years, and 20% (12/60) of patients reported low levels of ICT use. Moreover, we had to exclude patients with no ICT device or internet connection at home. For these reasons, we sought to develop a tool that is easy to use, even for patients who are not familiar with ICT use. We developed pocket-sized medication cards instead of digital cards for the same reasons, and patients appreciated these cards even more than the video. mHealth cannot replace the face-to-face approach, but it can be used as a complementary tool to increase patients’ self-efficacy. In this study, the pharmacist talked with patients and answered their questions after they had watched the video, which may have affected their satisfaction. We will continuously update the content and enhance the interactivity of our web-based video, and we plan to develop a complete app with an easily updated medication plan, a frequently asked questions module, and quizzes to help patients develop even more knowledge.

### Limitations

This study has some limitations. The ARMS is not the gold standard for the assessment of drug adherence; such assessment should include the direct measurement of drug consumption. Unfortunately, patients’ drug consumption cannot be determined accurately using data from the Swiss health care system, as drugs are delivered by packs containing a prespecified number of tablets (eg, ticagrelor is available in blister packs of 56 tablets, whereas aspirin is delivered by packs of 98 tablets) and are not delivered by drug unit (for example, 30 tablets for 30 days of treatment). In addition, we did not adjust for some baseline characteristics (eg, age, sex, and educational level), which may have resulted in bias. Nevertheless, all baseline characteristics were similar in the two study groups, which reduced the risk of statistical errors; we thus believe that the intervention had an impact on drug adherence. Another limitation is the small sample, which together with the high degree of variance resulted in the loss of statistical power and the inability to perform subgroup analyses. We had difficulties in recruiting patients because many of them were transferred within 24 hours to a peripheral hospital, and we lost several of them to follow-up. We unfortunately could not continue the recruitment further, and we had to analyze the results with the small sample. The exclusion of patients without home internet access also may have biased the results. Finally, our findings may have been affected by attrition bias, as we lost about 20% (13/60) of patients between baseline and study completion. Despite these limitations, our results are very encouraging and suggest that the provision of an interactive informational video to patients has positive effects. These results should be confirmed in larger clinical studies that include subgroup analyses to identify the populations that would benefit most from such interventions.

### Conclusions

Despite our study being underpowered, we were still able to show that the “Mon Coeur, Mon BASIC” web-hosted interactive video improved drug adherence and enhanced patients’ cardiovascular knowledge. The video will be available at no charge from a webpage constructed by our hospital’s cardiology department staff and will be offered to all patients hospitalized for ACS at our institution in the future. Our findings reflect patients’ need for and appreciation of medical information; the most appropriate means of providing such information needs to be determined. Such tools should be developed for patients with a wide range of chronic diseases, and their content should be continuously improved. Over time, increasing numbers of patients will be able to use smartphone apps, which provides the opportunity to develop this type of support to improve the management of patients with chronic diseases.

## Appendix

Multimedia Appendix 1 Medication card distributed to the intervention group, in addition to the video viewing.

Multimedia Appendix 2 ARMS score questionnaire developed by Kripalani et al, with the presentation of the 12 questions and his scoring system. ARMS: Adherence to Refills and Medication Scale.

Multimedia Appendix 3 Questionnaire used for the assessment of the patient's knowledge. Correct answers were evaluated with 1 point and wrong answers with 0 points. Maximal points were 9 points.

Multimedia Appendix 4 CONSORT-eHEALTH checklist (V 1.6.1).

## Abbreviations

ACS: acute coronary syndrome
ARMS: Adherence to Refills and Medication Scale
BASIC: Secondary Prevention of ACS With Beta-Blockers, Antiaggregants, Statins, Angiotensin-Converting Enzyme Inhibitors, and Risk Factor Control
CR: cardio-rehabilitation
FCCHL: Functional, Communicative and Critical Health Literacy
ICT: information and communications technology
mHealth: mobile health
MI: myocardial infarction
NSTEMI: non–ST-segment elevation myocardial infarction
PCI: percutaneous coronary intervention
REDCap: Research Electronic Data Capture
STEMI: ST-segment elevation myocardial infarction
