# Supplementary figures and images for: Effects of the Interactive Web-Based Video “Mon Coeur, Mon BASIC” on Drug Adherence of Patients With Myocardial Infarction: Randomized Controlled Trial
Source: J Med Internet Res. 2021 Aug 30;23(8):e21938. doi: 10.2196/21938 (PMC8438608; doi:10.2196/21938)

#
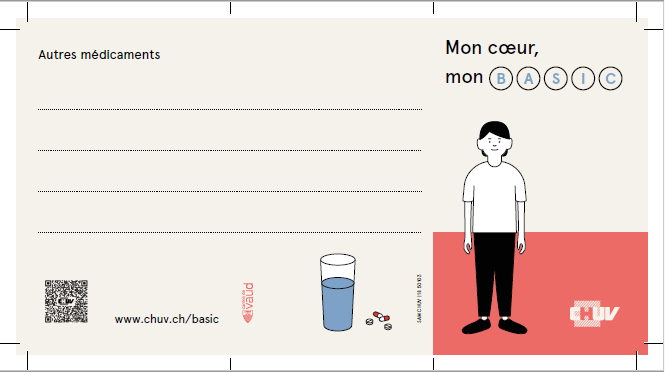
S1. Medication card


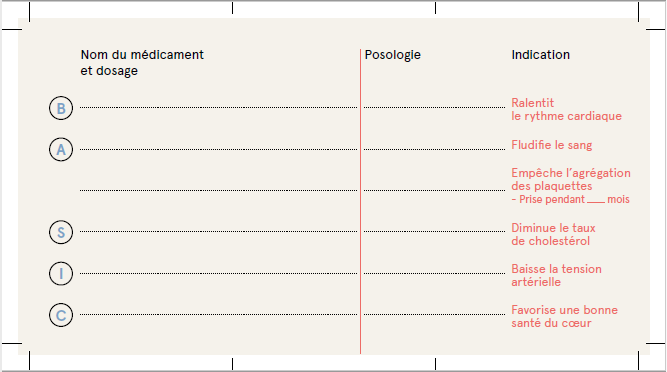

Supplement: Multimedia Appendix 1 [file jmir_v23i8e21938_app1.docx]
