# Supplementary material for: Effects of the Interactive Web-Based Video “Mon Coeur, Mon BASIC” on Drug Adherence of Patients With Myocardial Infarction: Randomized Controlled Trial
Source: J Med Internet Res. 2021 Aug 30;23(8):e21938. doi: 10.2196/21938 (PMC8438608; doi:10.2196/21938)
Supplement: Multimedia Appendix 3 [file jmir_v23i8e21938_app3.docx]

# S3. Knowledge questionnaire

| **Questionnaire de connaissances sur l’infarctus du myocarde et les médicaments** | | | |
| --- | --- | --- | --- |
| ID |  | Date |  |

**Ce questionnaire a pour but de savoir quelles sont vos connaissances sur votre maladie et sur sa prise en charge. Dans le cadre de l’étude BASIC, nous vous demandons de répondre aux questions suivantes (une seule réponse possible) :**

1. **Que sont les coronaires?**

- Des vaisseaux qui distribuent du sang oxygéné dans les organes du corps
- Des vaisseaux qui distribuent du sang oxygéné au cœur pour le faire fonctionner
- Des vaisseaux qui apportent du sang non-oxygéné du cœur vers les poumons
- Je ne sais pas

1. **Qu'est-ce que l'athérosclérose ?**

- Une maladie chronique qui comprend l'inflammation et le développement de plaques composées d'acide gras et de cholestérol dans la paroi des artères
- Une maladie aigue qui consiste en la rupture de plaque dans la paroi des artères et la formation d’un caillot de sang
- Une maladie chronique du système sanguin où le sang devient trop épais et obstrue les artères qui irriguent le cœur
- Je ne sais pas

1. **Que se passe-t-il au moment d'un infarctus du myocarde ?**

- Le cœur se fatigue progressivement et, suite à un effort ou un stress, il s'arrête de battre subitement
- Une plaque d’athérome se rompt dans une coronaire, un caillot se forme et bloque le passage de sang oxygéné dans une partie du cœur
- Le muscle du cœur se fragilise progressivement, il se rompt et laisse s’écouler le sang dans le corps
- Je ne sais pas

1. **A quoi sert un stent?**

- A garder la coronaire bien ouverte
- A empêcher la formation d'un caillot de sang dans les coronaires
- A diminuer la progression des plaques d’athérome dans les coronaires
- Je ne sais pas

1. **Quels sont les médicaments généralement prescrits après un infarctus?**

- Bétabloquant, Aspirine et antiagrégant, Statine et Inhibiteur de l’enzyme de conversion (IEC)
- Anticalciques, Statines, Inhibiteur de la pompe à proton (IPP), Analgésique
- Anticoagulant, Inhibiteur de l’enzyme de conversion (IEC), inhibiteur de la pompe à proton (IPP)
- Je ne sais pas

1. **Que devez-vous faire si vous ressentez à nouveau des douleurs thoraciques qui ne passent pas après 15 minutes ?**

- J’appelle mon médecin traitant
- Je me rends aux urgences
- J’appelle une ambulance
- Je ne sais pas

1. **A quoi servent l'aspirine (Aspirine cardio^®^) et l'antiagrégant (Brilique®, Plavix®, Clopidogrel ou Efient^®^) ?**

- A diminuer le taux de cholestérol
- A fluidifier le sang
- A diminuer la pression artérielle
- Je ne sais pas

1. **Combien de temps doit-on généralement prendre l'aspirine (Aspirine cardio^®^) et l'antiagrégant (Brilique®, Plavix®, Clopidogrel ou Efient^®^)** ?

- Tous les deux à vie
- L'antiagrégant à vie et l'aspirine une année
- L'aspirine à vie et l'antiagrégant une année
- Je ne sais pas

1. **A quoi servent les statines (Atorvastatine, Pravastatine, Sortis^®^, Livazo^®^, Inegy^®^)**?

- A diminuer le taux de cholestérol
- A fluidifier le sang
- A diminuer la pression artérielle
- Je ne sais pas
